# Anaerobic slurry co-digestion of poultry manure and straw: effect of organic loading and temperature

**DOI:** 10.1186/2052-336X-11-15

**Published:** 2013-07-03

**Authors:** Azadeh Babaee, Jalal Shayegan, Anis Roshani

**Affiliations:** 1 University of Sharif, Tehran, Iran; 2 Azad University of Tehran, Tehran, Iran

**Keywords:** Anaerobic digestion, Poultry manure, Wheat straw, Biogas, Yield

## Abstract

In order to obtain basic design criteria for anaerobic digestion of a mixture of poultry manure and wheat straw, the effects of different temperatures and organic loading rates on the biogas yield and methane contents were evaluated. Since poultry manure is a poor substrate, in term of the availability of the nutrients, external supplementation of carbon has to be regularly performed, in order to achieve a stable and efficient process. The complete-mix, pilot-scale digester with working volume of 70 L was used. The digestion operated at 25°C, 30°C and 35°C with organic loading rates of 1.0, 2.0, 2.5, 3.0, 3.5 and 4.0 kg Volatile solid/m^3^d and a HRT of 15 days. At a temperature of 35°C, the methane yield was increased by 43% compared to 25°C. Anaerobic co-digestion appeared feasible with a loading rate of 3.0 kg VS/m^3^d at 35°C. At this state, the specific methane yield was calculated about 0.12 m^3^/kg VS with a methane content of 53–70.2% in the biogas. The volatile solid (VS) removal was 72%. As a result of volatile fatty acid accumulation and decrease in pH, when the loading rate was less than 1 or greater than 4 kg VS/m^3^d, the process was inhibited or overloaded, respectively. Both the lower and higher loading rates resulted in a decline in the methane yield.

## Introduction

In the past few decades, the large amounts of animal manure and slurries have been produced by the animal breeding sector as well as the wet organic waste streams represent a constant pollution risk with a potential negative impact on the environmental, if not managed optimally. When untreated or poorly managed, animal manure becomes a major source of air and water pollution. Nutrient leaching, mainly nitrogen, phosphorus and ammonia evaporation and pathogen contamination are some of the major threats [1]. The animal production sector is responsible for 18% of the overall greenhouse gas emissions, measured in CO_2_ equivalent and for 37% of the anthropogenic methane, which has 23 times the global warming potential of CO_2_[2]. Annually, approximately 400 million tons of wastes have been produced by Iran livestock industry (cattle and poultry) and agriculture sector. It means that it really needs an integrated waste management.

Anaerobic co-digestion of organic matters results in waste stabilization as well as in biogas production. This gas usually contains more than 50% methane, and therefore it can be used as bio-fuel in power generation systems to produce heat and energy [3]. Wastes have been effectively used as biogas materials by various studies [4]. The other Benefits of the anaerobic digestion of animal manure are pathogen reduction through sanitation, improved fertilization efficiency, less nuisance from odors and flies and etc. [5]. Anaerobic digestion reduces the majority of pathogenic agents, if can be carried out under mesophilic or thermophilic conditions [6].

The anaerobic digestion of organic material is a complex process, involving a number of different degradation steps. The microorganisms that participate in the process may be specific for each degradation step and thus could have different environmental requirements such as temperature, pH, moisture, carbon source, nitrogen and C/N ratio. Many researchers have reported significant effects of temperature on the microbial community, process kinetics and stability and methane yield. Lower temperatures during the process are known to decrease microbial growth, substrate utilization rates, and biogas production. Moreover, lower temperatures may also result in an exhaustion of cell energy, a leakage of intracellular substances or complete lysis. In contrast, high temperatures lower biogas yield due to the production of volatile gases such as ammonia which suppresses methanogenic activities [7].

Animal waste often has very high total ammonia nitrogen concentrations due to presence of ammonia as well as protein and urea [8]. Nitrogen is an essential nutrient for anaerobic organisms [9], consequently, the inhibitory effects of ammonia, as far as is known, influence mainly the phase of methanogenesis in anaerobic reactors [10], released from decomposition of organic ammonia. It has been suggested that poultry manure is best treated with other wastes because of its high nitrogen content [11]. Crop residues represent another fraction of agricultural waste. Substantial quantities of unused stalks, straws and bark are produced from a variety of crops, which could be used for energy generation, but they are poor substrate in term of nitrogen and phosphate. Therefore, co-digestion of animal manure and crop residues can supply a proper C/N ratio for microorganisms. This ratio is the balance of food that a microbe requires in order to grow. The optimal C/N ratio is 20–30 and excess N can lead to ammonia inhibition of digestion [12]. The unbalanced nutrients are regarded as an important factor limiting anaerobic digestion of organic wastes. For the improvement of nutrition and C/N ratios, co-digestion of organic mixtures is employed.

In spite of high production of poultry manure, anaerobic digestion of this kind of organic waste has not been studied as much as cow and swine manure. Cow manure is a cellulose-rich component that high amount of cellulose and hemi- cellulose cause suitable C/N ratio and therefore it can be digested easily in anaerobic conditions. It is also a good fertilizer as it will not burn the plants. Inversely, poultry manure is an ammonia-rich component which cannot be used as a fertilizer because it will burn the plants as a result of high ammonia content. In order to obtain suitable C/N ratio, co-digestion of poultry manure has been done with hog manure, swine manure, fruit and vegetable wastes [13]. Co-digestion can utilize the nutrients and bacterial diversities in various wastes to optimize the digestion process. Co-digestion of poultry manure with wheat straw that has been done in this study may be considered as a new issue and the objective of the study is finding the optimum temperature and loading rate in a pilot-scale reactor that will be discussed fully below.

## Material and methods

### Waste characteristics

The poultry waste and straw were obtained from a local farm in Tehran and stored at 4°C. Poultry manure and straw were mixed together, straw with portion of 80% by weight and Poultry manure 20% by weight in order to supply a proper C/N ratio (C/N≈23). The waste mixture consist of 90% total solid (TS) and 80% of TS was Volatile Solid. The other characteristics of waste are presented in Table 1. Components of the obtained samples were determined by the procedures described in the Standard Methods [14].

**Table 1:** Characteristics of the feed solids as sampled

**Parameters**	**Poultry manure**	**Straw**
Ammonia nitrogen (w/w %)	5.65	0.61
Total Nitrogen (w/w %)	5.67	0.63
COD (w/w %)	35.88	51.88
C/N ratio	6.35	84.22
pH	7.3	-----

### Experimental set-up

A cylindrical CSTR reactor with a working volume of 60 L (total digester volume 70 L) was operated at a 15d HRT for all runs. The reactor was fitted with a top plate, which supported the mixer, mixer motor and gas sampler. Sampling valves were located at positions corresponding to the top, middle and bottom layer of digester contents. The reactor had one outlet at the bottom for effluent removal. The contents of the reactor were mixed as controlled by a timer, which was activated for 30 min every hour. It was operated at 35°C and then was obtained at 30°C and 25°C.

### Reactor operation

First, 40 L of anaerobic sludge from a dairy factory and 20 L water were transferred to the reactor. Daily feeding was commenced approximately 24 days after start-up. Raw waste characteristics over the study period are given in Table 1. The digestion operated at 25°C, 30°C and 35°C with organic loading rates of 1.0, 2.0, 2.5, 3.0, 3.5 and 4.0 kg VS/m^3^d. For preventing accumulation, because of daily feeding, 4 liters of content was removed every day. It was calculated according to HRT of 15 days. It is worth mentioning that gradual hydrolysis of cellulose caused the high amount of COD accumulation.

### Analytical methods

The produced biogas was measured daily by water displacement method and its composition was measured by gas chromatograph. Total solids (TS), volatile solids (VS), pH and alkalinity were determined according to the APHA Standard Methods [14]. Total nitrogen (TN) was estimated by the Kjeldahl method [14].

## Result

### Effect of temperatures and feed loads on the methane yield

**Figure 1:**
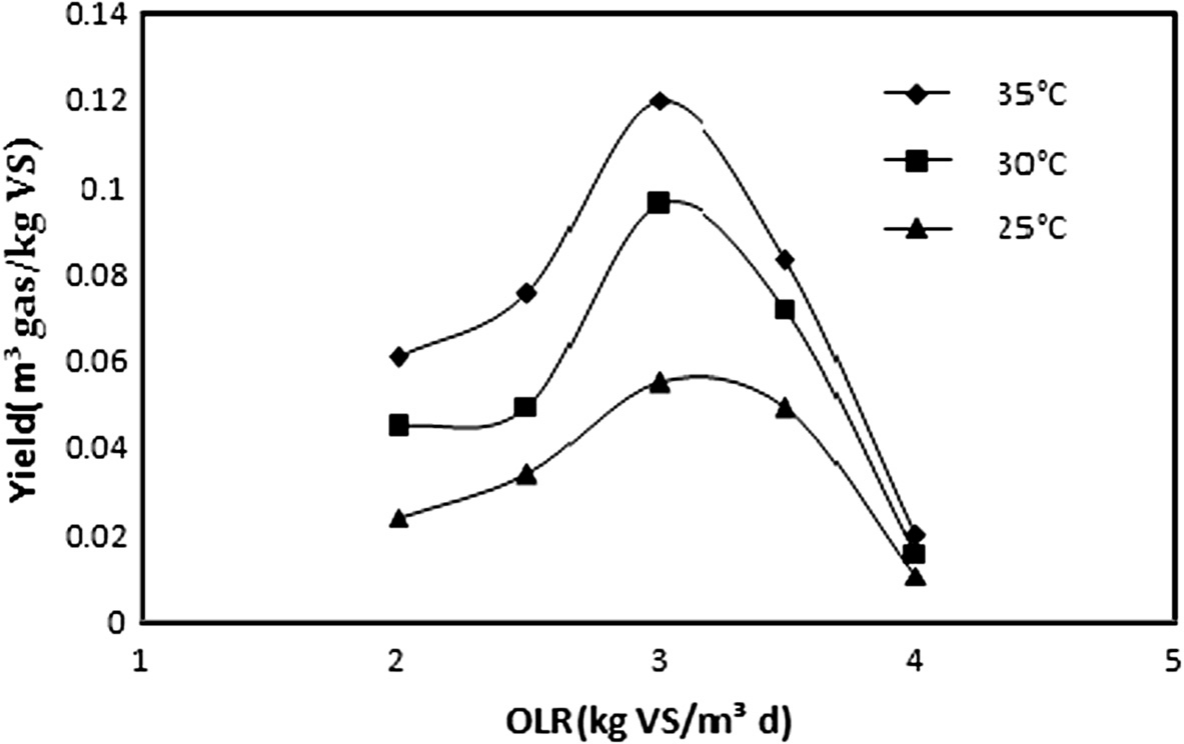
Methane yield of different loads and temperatures.

The influence of temperatures and feed loads on the methane yield is shown in Figure 1. Regardless of the temperature, increasing the feed load from 2.0 to 3.0 kg VS/m^3^d increased the methane yield and after that, a gradual increase in the OLR caused a decrease in the methane yield. According to the results, total gas production reached the highest amount at 35°C for a loading rate of 3.0 kgVS/m^3^d (0.12 m^3^CH_4_/kgVS) and methane yield increased (43% higher at 35°C relative to 25°C) with a temperature increase from 25 to 35°C. At 35°C, ultimate methane yield of 0.061, 0.12 and 0.02 m^3^CH_4_/kg VS were obtained at 2.0, 3.0 and 4.0 kgVS/m^3^d, respectively.

### Effect of temperatures and feed loads on biogas composition

**Figure 2:**
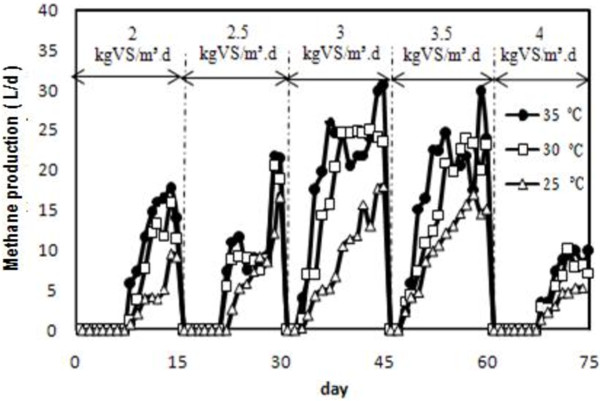
Variation of methane production in different loads and temperatures.

One of the main objectives of this research was to determine production and composition of biogas during anaerobic process at different loading rates and temperatures. The biogas production at steady-state condition was found to be 44.8, 36 and 26 L/d at a load of 3.0 kg VS/m^3^d at 35, 30 and 25°C, respectively. Further increase of the feeding rate up to 4.0 kg VS/m^3^d resulted in decreases in biogas production rates. The variation of methane production is shown in Figure 2. The maximum methane contents (70.2%) were calculated at a load of 3.0 kg VS/m^3^d (35°C) and the minimum (30%) was at a load of 4.0 kg VS/m^3^d (25°C).

### Effect of temperatures and feed loads on COD accumulation

**Figure 3:**
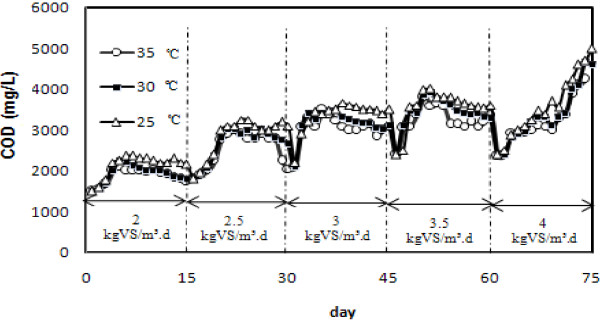
Variation of COD in different loads and temperatures.

Population of anaerobic microorganisms and their adaptation to the new environmental conditions typically take a significant period of time to establish themselves to be fully effective. According to Figure 3, a small quantity of methane production at the first load allowed for accumulation of soluble COD up to 3010 mg/L at 2.5 kg VS/m^3^d and ascending rates of methane production were observed. After a gradual increase of OLR up to 3.0 kg VS/m^3^d, methane production increased up to 31.4 L/d and COD reached 3200 mg/L. VS removal was calculated 72% at this load but it decreased at other loads. In the next loads of 3.5 and 4.0 kg VS/m^3^d COD increased up to 3800 and 4900 mg/L, respectively (at pH= 7.5 and 7.1, free ammonia concentration 240 and 261 mg/L). The process appeared inhibited and/or overloaded by the accumulation of volatile fatty acids that lead methanogens to be inactive. The results at 30 and 35°C were similar.

### Effect of temperatures and feed loads on pH

**Figure 4:**
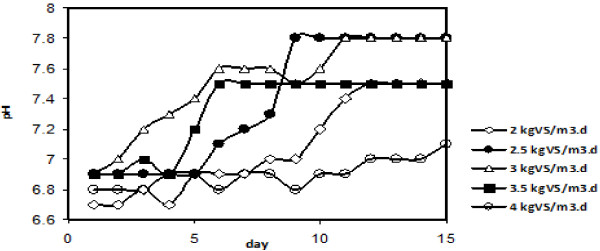
The variation of pH at 35°C.

The pH variation at 35°C is shown in Figure 4. The pH stabilized between 6.8±0.1 and 7.8±0.1 in all runs. Hence the pH, which did not vary significantly among different loads, was in the optimum range for methanogens in all treatments. Total NH_3_-N within each load did not vary a lot, and ranged from 68 to 256 mg/L (Figure 4).

## Discussion

### Effect of temperatures and feed loads on the methane yield

The loading rate and temperature are obviously critical process parameters in anaerobic treatment. The influence of temperature and feed loads on the methane yield have been shown in Figure 1. These findings are in agreement with the results of Alvarez et al. [15]. They reported that there was a linear relationship between methane yield and loading rate at lower loading rates. The maximum methane yield was observed at 35°C (0.12 m^3^CH_4_/kgVS) because of the suitable type and composition of substrate, microbial composition and temperature. At intermediate loading rates, methane yield was almost constant up to a certain loading at which it starts to decrease. This breakpoint indicates the beginning of biological stress and beyond this point, the methane production rate decreased sharply. At 30°C, the yield was 0.045, 0.096 and 0.0157 m^3^CH_4_/kgVS for loads of 2.0, 3.0 and 4.0 kg VS/m^3^d, respectively that was lower than the yields at 35°C. Callaghan et al. [11] have reported the methane yield of 0.23 m^3^/kgVS and 50% VS reduction by using a co-digestion system of fruit and vegetable waste (FVW) and Chicken manure with HRT of 21 days at 35°C. According to Salminen et al. [16], the potential methane yield of solid poultry slaughterhouse waste and HRT of 50–100 days at 31°C is high, from 0.52 to 0.55 m^3^/kg VS. The treatment of wastewaters containing high levels of TS or indigestion components, such as slaughterhouse wastewater or straw, will require a longer reaction period for complete degradation of particles, especially at lower temperatures. It is the most important reason of low methane yield in this case study compared with the others’ results. Design parameters and related data are presented in Table 2.

**Table 2:** Average characteristics of mixture of poultry manure and straw and the effluents

**Parameter (in mg/ except pH &C/N)**	**Operating temperation (c)**																	
	**35**						**30**						**24**					
	**2**		**3**		**4**		**2**		**3**		**4**		**2**		**3**		**4**	
**OLR (kgVs/m**^**2 **^**d)**	**Inf**	**Eff**	**Inf**	**Eff**	**Inf**	**Eff**	**Inf**	**Eff**	**Inf**	**Eff**	**Inf**	**Eff**	**Inf**	**Eff**	**Inf**	**Eff**	**Inf**	**Eff**
COD	1185	1710	1783	3200	2360	4900	1185	1780	1783	3140	2360	5000	1185	2110	1783	3300	2360	5100
TS	2275	1153	3408	1482	4550	2616	2275	1100	3408	2010	4550	2010	2275	2000	3408	2700	4550	4020
VS	2000	750	300	840	4000	2290	2000	863	3000	900	4000	2400	2000	1048	3000	1170	4000	2440
Total nitrogen	ND^a^	256	ND	240	ND	261	ND	192	ND	180	ND	235	ND	160	ND	68	ND	105
pH	ND	7.5	ND	7.8	ND	7.5	ND	7.8	ND	7.8	ND	7.8	ND	7.0	ND	6.8	ND	6.7
Alkalinity	ND	2100	ND	2980	ND	3000	ND	2200	ND	2100	ND	3200	ND	3800	ND	4000	ND	4000
C/N	23.09		23.7		23.4													

^a^Not determined.

### Effect of temperatures and feed loads on biogas composition

Decreases in the methane content indicated hydraulic and organic overload and insufficient buffering capacity in the digester that they led to a reduction in the methanogenic activity. Chae et al. [17] reported that the biogas composition differed according to digestion temperature. Methane contents in the biogas were 65.3%, 64.0% and 62.0% at 35°C, 30°C and 25°C, respectively.

### Effect of temperatures and feed loads on COD accumulation

Decrease in the temperature had a negative effect on the metabolic rate of the microorganisms. For this reason, at 25°C COD increased sharply. Once 5100 mg/L, was reached, methane production decreased by 40% and VS removal dropped to 39%. In fact, the effect of temperature on organic removal rate did not seem to be uniform over the whole temperature spectrum. Chae et al. [17] reported that the digestion yield at a temperature of 25°C showed 82.6% of that at 35°C. These results were in agreement with previous results that showed an improvement in the biogas yields with increasing temperatures [18].

### Effect of temperatures and feed loads on pH

The pH stabilized between 6.8±0.1 and 7.8±0.1 in all runs. Both total and free ammonia concentration varied a bit between stages. A wide range of inhibiting ammonia concentrations has been reported in the papers the amount of ammonia in the digester may also affect the production of hydrogen and removal of volatile solids. Total biogas production was unaffected by small increases in ammonia nitrogen while higher increases reduced the biogas production by 50% of the original rate. In the fluidized-bed anaerobic digester, the methane formation decreased at ammonium concentrations of greater than 6000 mg NH4–N/L. It was reported that methanogenic activity is decreased by 10% at ammonium concentrations of 1670–3720 mg NH4–N/L, while by 50% at 4090–5550 mg NH4–N/L, and completely zero at 5880–6000 mg NH4–N/L [19].

The free NH_3_±N concentrations calculated in this study were far below those reported as inhibitory because of a) dilution of digester content with water and b) adjustment of feed C/N ratio. It should also be noted that both methanogenic and acidogenic microorganisms have their optimal pH. There is a considerable potential of biogas production from anaerobic digestion of poultry manure that offers several environmental, agricultural and socio-economic benefits throughout biogas production as a clean and renewable fuel. The process worked well with a loading of 3.0 kgVS/m^3^d VS removal amounted 72%. The temperature had an influence on the ultimate methane yield, as well as the methane contents. The highest temperature caused the most methane yield (0.12 m^3^/kg VS); however, the yield did not linearly increase with increasing temperature. More amount of methane yield may be achieved by extension of the hydraulic residence time because of the high levels of TS in waste.

## Conclusion

The study clearly indicates that anaerobic digestion is one of the most effective biological processes to treat a wide variety of solid organic waste products. The prime advantages of this technology include (i) organic wastes with a low nutrient content can be degraded by co-digesting with different substrates in the anaerobic bioreactors, and (ii) the process simultaneously leads to low cost production of biogas, which could be vital for meeting future energy-needs. However, different factors such as substrate and co-substrate composition and quality, environmental factors (temperature, pH, organic loading rate), and microbial dynamics contribute to the efficiency of the anaerobic digestion process, and must be optimized to achieve maximum benefit from this technology in terms of both energy production and organic waste management. This technology has tremendous application in the future for sustainability of both environment and agriculture, with the production of energy as an extra benefit.

## Competing interests

The authors declare that they have no competing interests.

## Authors’ contributions

AB participated in the conception of the study, interpretation of data and in the given final approval of the version to be published; JSH supervised the study in all steps (acquisition, analysis, and interpretation of data); AR participated in the acquisition, analysis, and interpretation of data and helped to draft the manuscript. All authors read and approved the final manuscript.

## References

[B1] Oleskowicz-Popiel P Seadi TA Holm-Nielsen JB The future of anaerobic digestion and biogas utilization Bioresour Technol 2009 100 5478 5484 10.1016/j.biortech.2008.12.046 19217772

[B2] Steinfeld H Gerber P Wasenaar T Castel V Rosales M de Haan C Livestock’s long shadow 2006 Food and Agriculture Organization (FAO) of United Nations: Environmental issues and Options

[B3] Ojolo SJ Oke SA Animasahun K Adesuyi BK Utilization of poultry, cow and kitchen wastes for biogas production: comparative analyses Iranian Journal of Environmental Health Science Engineering 2007 4 Suppl 4 223 228

[B4] Ojolo SJ Bamgboye AI Ogunsina BS Oke SA Analytical approach for predicting biogas generation in a municipal solid waste anaerobic digester Iranian Journal of Environmental Health Science Engineering 2008 5 Suppl 3 179 186

[B5] Sommer SG Moller HB Petersen SO Reduction in methane and nitrous oxide emission from animal slurry trough anaerobic digestion Proceedings of the Third International Symposium 2002 Maastricht, Netherlands: Millpress Science Publisher 475 480

[B6] Takdastan A Movahedian H Jafarzadeh N Bina B The Efficiency of Anaerobic Digesters on Microbial Quality of Sludge in Isfahan and Shahinshahr Waterwaste Treatment Plant Iranian Journal of Environmental Health Science Engineering 2005 2 Suppl 1 56 59

[B7] Khalid A Arshad M Anjum M Mahmood T Dawson L The anaerobic digestion of solid organic waste Waste Manag 2011 31 1737 1744 10.1016/j.wasman.2011.03.021 21530224

[B8] Zeeman G Wiegant WM Koster-Treffers ME Lettinga G The influence of the total ammonia concentration on the thermophilicdigestion of cow manure Agricultural Wastes 1985 14 19 35 10.1016/S0141-4607(85)80014-7

[B9] Strik DPBTB Domnanovich AM Holubar PA A pH-based control of ammonia in biogas during anaerobic digestion of artificial pig manure and maize silage Process Biochem 2006 41 1235 1238 10.1016/j.procbio.2005.12.008

[B10] Calli B Mertoglu B Inanc B Yenigun O Effects of high free ammonia concentrations on the performances of anaerobic bioreactors Process Biochem 2005 40 1285 1292 10.1016/j.procbio.2004.05.008

[B11] Callaghan FJ Wase DA Thayanithy K Forster CF Continuous Co-digestion of cattle slurry with fruit and vegetable wastes and chicken manure Journal of Biomass & Bioenergy 2002 27 71 7724052568

[B12] Molnar L Bartha I High solids anaerobic fermentation for biogas and compost production Biomass 1998 16 173 182

[B13] Johnston PH Adams TT Magbanua J Anaerobic co-digestion of hog and poultry waste Bioresour Technol 2001 76 165 168 10.1016/S0960-8524(00)00087-0 11187082

[B14] APHA Standard methods for the examination of water and wastewater 1998 20 Washington, DC: American Public Health Assoc

[B15] Alvarez R Liden G Semi-continuous co-digestion of solid slaughterhouse waste, manure, fruit and vegetable wastes Renew Energy 2008 33 726 734 10.1016/j.renene.2007.05.001

[B16] Salminen EA Rintala J Semi-continuous anaerobic digestion of solid poultry slaughterhouse waste: Effect of hydraulic retention time and loading Water Res 2002 36 3175 3182 10.1016/S0043-1354(02)00010-6 12188113

[B17] Chae KJ Jang A Kim IS Yim SK The effect of digestion temperature and temperature shock on the biogas yields from the mesophilic anaerobic digestion of swine manure Bioresour Technol 2008 99 1 6 10.1016/j.biortech.2006.11.063 17306978

[B18] Masse DI Masse L The effect of temperature on slaughterhouse wastewater treatment in anaerobic sequencing batch reactors Bioresour Technol 2001 76 91 98 10.1016/S0960-8524(00)00105-X 11131805

[B19] Sawayama S Tada C Tsukahara K Yagishita T Effect of ammonium addition on methanogenic community in a fluidized bed anaerobic digestion Journal of Bioscience Bioenergy 2004 97 65 70 10.1016/S1389-1723(04)70167-X 16233591

